# SGGformer: Shifted Graph Convolutional Graph-Transformer for Traffic Prediction

**DOI:** 10.3390/s22229024

**Published:** 2022-11-21

**Authors:** Shilin Pu, Liang Chu, Jincheng Hu, Shibo Li, Jihao Li, Wen Sun

**Affiliations:** 1 College of Automotive Engineering, Jilin University, Changchun 130022, China; 2 Department of Aeronautical and Automotive Engineering, Loughborough University, Loughborough LE11 3TU, UK; 3 College of Automotive Engineering, Changzhou Institute of Technology, Changzhou 213032, China

**Keywords:** Graph Transformer, multi-channel GCN, shifted window operation, traffic prediction, deep learning

## Abstract

Accurate traffic prediction is significant in intelligent cities’ safe and stable development. However, due to the complex spatiotemporal correlation of traffic flow data, establishing an accurate traffic prediction model is still challenging. Aiming to meet the challenge, this paper proposes SGGformer, an advanced traffic grade prediction model which combines a shifted window operation, a multi-channel graph convolution network, and a graph Transformer network. Firstly, the shifted window operation is used for coarsening the time series data, thus, the computational complexity can be reduced. Then, a multi-channel graph convolutional network is adopted to capture and aggregate the spatial correlations of the roads in multiple dimensions. Finally, the improved graph Transformer based on the advanced Transformer model is proposed to extract the long-term temporal correlation of traffic data effectively. The prediction performance is evaluated by using actual traffic datasets, and the test results show that the SGGformer proposed exceeds the state-of-the-art baseline.

## 1. Introduction

Intelligent transportation system (ITS) shows great potential in improving the efficiency [1], stability, and reliability of transportation systems, which makes it a research hotspot [2]. The development of ITS is mainly reflected in vehicle–environment coordination control [3], passenger demand forecasting [4], travel time estimation [5], and order scheduling [6]. The realization of these functions depends on accurate and reliable traffic forecasting. Furthermore, the improvement of computing power and data acquisition technology provides hardware and data support for the traffic prediction task, making the development of accurate traffic prediction methods a hot research topic.

The research history of scholars on traffic prediction methods mainly includes statistical, shallow machine learning, and deep learning methods. The earliest statistical methods used to predict traffic by modeling the nonlinear characteristics of the time series of traffic data include ARIMA, Gaussian regression, and Bayesian network [7,8,9]. These methods rely heavily on the periodicity of a single series and lack the ability to model the spatial correlation characteristics of traffic data, so their potential for traffic forecasting needs to be improved. Basic machine learning, such as SVM and ANN, can capture traffic data’s spatial and nonlinear temporal correlation [10,11], and its prediction effect depends heavily on professional feature engineering methods. Because there is no universally recognized and practical feature selection guide for different problems, basic machine learning-based methods may not maximize actual performance in the face of complex prediction tasks. Therefore, to capture more abundant and deeper spatiotemporal correlation information to bring more performance improvement to traffic prediction, the deep learning methods provide a solution for this problem.

Deep learning provides different solutions for feature extraction of spatial and temporal correlations. In the task of spatial correlation acquisition, the early applied methods focus more on convolutional neural networks (CNNs). Thanks to local connectivity and weight-sharing characteristics of CNNs, it has a compelling feature extraction performance for traffic data in the European space [12,13,14]. However, with the in-depth study of traffic data and the development of graph neural networks (GNNs), the definition of graph networks is more consistent with the non-European characteristics of the spatial distribution of traffic flow, which cause CNNs to become difficult to adapt to the definition form of these data. Graph Convolutional Networks (GCNs) have become more mainstream spatial correlation feature extraction methods because they can capture the non-Euclidean correlation between graph network nodes [15,16,17,18]. The way to extract spatial correlation by GCNs has become the focus of research. Among them, Kipf et al. proposed the Chebyshev first-order approximate Graph Convolutional Network (GCN) to balance the performance of feature extraction and computational complexity and it became the mainstream GCN method [19], which was used in traffic prediction by a large number of movements. For example, STGCN, DCRNN, STSGCN, and other excellent models have been proposed [20,21,22,23,24], and GCN is adopted to complete the correlation feature extraction of the complex spatial domain.

When it comes to the capture task of nonlinear time correlation in traffic data, the early commonly used methods are recurrent neural networks (RNN) and their variants, Long Short-Term Memory neural network (LSTM), and Gated Recurrent Unit (GRU). The advantage of these methods is that they can retain sufficient historical information and discard useless information through recursive neurons, which leads to their application by many people to capture traffic temporal characteristics [25,26,27,28]. However, RNN-like methods tend to forget adequate long-term information for long-term sequences, leading to performance degradation [29]. Then, with the breakthrough progress in natural language processing (NLP), the model based on the Attention mechanism has become a research hotspot in capturing temporal correlation, which shows better performance in capturing long-term correlation. The attention mechanism can reduce the maximum length of the propagation path of the network signal to the theoretical shortest O (1) while avoiding the recursive structure, increasing the calculation efficiency [30]. Bai et al. introduced the attention mechanism to adjust the importance of different time points and integrated the global temporal information to improve prediction accuracy [31]. Guo et al. proposed the ASTGCN based on the attention mechanism to effectively capture the dynamic spatiotemporal correlation in traffic data by establishing the spatiotemporal attention mechanism [32]. Xu et al. dynamically modeled directional spatial dependencies by using a self-Attention mechanism to capture real-time traffic flow. Then, the temporal attention mechanism is adopted to model long-term time dependency across multiple time steps [29]. These advanced methods use the Attention mechanism in the Transformer to extract the correlation features of time series or spatial series and achieve good results. However, they do not make good use of the Seq2Seq architecture in the Transformer model. The advantage of Transformer architecture is that it separates the learning of sequence information from the prediction of future information through the encoder–decoder structure rather than doing it simultaneously as a simple RNN-type network, which increases the network’s learning ability and supports the prediction of any length at the same time.

Transformer, as a revolutionary innovation based on the Attention mechanism [33], integrates the self-attention mechanism into the Seq2Seq architecture to further increase the time-related capture capability and becomes one of the future research directions. TSTNet combines GAT with a Transformer based on Seq2Seq architecture to extract the spatiotemporal correlation characteristics [34]. The transformer uses graph context embedding to encode the input temporal and spatial information. It then embeds the target node, neighbor node, and discrete time through the full connection layer into the input Transformer to find the correlation between different elements. This kind of exploration of the Transformer method is significant. Although it uses the Graph Attention Network (GAT) to learn some graph space information, it uses the embedding and concatenation of spatial features and temporal features as the input in the Transformer. Specifically, the spatial and temporal information are regarded as elements in the sequence, which is different from simply using a Transformer as a time modeling device, which may result in some loss of spatial topology information.

To fully extract the spatiotemporal correlation characteristics of traffic data, this paper inspired by advanced work of T-MGCN and Informer [30,35] proposes a model combining a shifted window operation, GCN, and Graph Transformer. This model uses a shifted window operation to coarsen time segments to reduce fluctuation noise and computational complexity; Multi-channel GCN is used to fuse spatial correlation based on different features; the improved Graph Transformer can directly extract the nonlinear time correlation characteristics of high-dimensional graph network data. Finally, the progressiveness of this method is verified by comparison with the classic baseline. The main contributions of this paper are as follows:

1. This paper constructs a prediction network that integrates a shifted window operation, GCN, and Transformer models, namely Shifted Graph Convolutional Graph-Transformer (SGGformer). GCN realizes complex spatial correlation characteristics extraction, and Graph Transformer extracts the nonlinear temporal correlation characteristics;
2. The shifted window operation is developed to divide time segments, reduce computational complexity, and enhance the ability to capture features of different periods;
3. The regional relationship with the graph network is defined, and the aggregation and mapping of regional nodes under the definition of a topology network is completed based on a multi-channel GCN;
4. The improved Graph Transformer is used to process high-dimensional graph data. The generative decoder outputs long sequences in a single step to avoid cumulative errors and significantly reduce reasoning speed.

The rest of the paper is organized as follows: In Section 2, the deep learning method for extracting spatial and temporal correlation features of traffic is introduced. In Section 3, the definitions related to traffic prediction and the structure of the SGGformer model proposed in this paper are introduced, respectively. In Section 4, data preprocessing, evaluation index establishment, comparison test with baseline, and ablation analysis are described. Then, a discussion of the results of the experiments is presented in Section 5. Finally, conclusions are drawn with future study directions in Section 6.

## 2. Related Works

This section outlines the existing methods for modeling spatial and temporal dependencies in traffic flow prediction. Because statistical and traditional machine learning methods cannot effectively model spatial dependencies, these methods are mainly based on deep learning models.

### 2.1. Spatial Correlation Extraction

CNNs are the first depth learning method used in traffic prediction. Because of its local perception and weight-sharing characteristics, it is widely used in traffic prediction. Toncharoen et al. used a convolutional neural network (CNN) to extract the spatial characteristics of node data along the highway [12]. Yao et al. used a CNN model to capture the spatial characteristics of traffic data distributed in regional form [13]. Wang et al. used the 3D-CNN and sparse UNet method to model the spatial correlation of traffic data [14]. Cao et al. extracted the characteristics of the target road and the surrounding roads with strong correlation through CNN [36]. However, this kind of network is more suitable for the task of a regular network, which will cause the loss of topological information in the face of an irregular traffic network. Thanks to the robust feature extraction ability of graph neural networks (GNNs) for graph information, traffic prediction is extended to the non-European domain. As a variant of GNNs, the GCN extends classical CNN to the graph domain. Recently, GCN has been widely used to model the non-European spatial correlation of traffic data, thus serving the traffic prediction task. Li et al. proposed the DCRNN which uses diffusion convolution on the directed graph to capture the diffusion information of traffic flow [21]. Yu et al. proposed the STGCN which uses spectral graph convolution on an undirected graph to model spatial correlation [20]. Guo et al. combined a spectral graph convolutional network and a temporal CNN to complete the extraction of temporal and spatial correlation [32]. Song et al. proposed the spatiotemporal synchronous graph convolution network STSGCN, and built a spatiotemporal synchronous modeling mechanism to effectively capture the complex local spatiotemporal correlation, in which the local spatial correlation extraction is completed through the superposition of multiple graph convolution operations [22]. Therefore, it can be found that the current development direction for spatial correlation extraction is based on a graph convolution network, and the difference lies in the construction method of the adjacency matrix. A reasonable construction can effectively balance the computational efficiency with the performance of spatial feature capture.

### 2.2. Temporal Correlation Extraction

RNN is the earliest method used to extract the time correlation of traffic data. Because gradient explosion, gradient disappearance, and other problems often occur, RNN has limitations in modeling time correlation. In order to overcome these problems, LSTM and GRU are used to build the long-term dependency relationship in the traffic sequence. Xiao et al. used a two-way LSTM (Bi-LSTM) model to extract the periodic characteristics in the daily and weekly traffic data and used the two-way characteristics of LSTM to capture the forward and backward traffic flow change trends [25]. Tian et al. used the LSTM model to effectively capture the complex temporal-related characteristics of traffic flow in the short-term traffic flow prediction task [26]. However, these models are all based on recursive processes, and there are always problems such as training, time-consumption, and long sequence information forgetting. Some scholars tried to model the temporal correlation of sequences through CNN [20,32,37]. However, its receptive field range could not meet the requirements of long-term sequence input, and its scalability was constrained as the number of hidden layers increased with the increase of sequence length. With the breakthrough and development of the NLP field, research based on the Transformer model has become a hot spot. The Attention mechanism captures the temporal correlation in the transportation field due to its strong ability to extract the correlation of sequence elements. Yao et al. proposed a new spatiotemporal dynamic network, introduced CNN to learn the dynamic similarity between regions, and designed a periodic attention-shifting mechanism to capture long-term periodicity [13]. Bai et al. used GRU to capture short-term trends and introduced attention mechanisms to adjust the importance of different time points which integrated global time information to improve prediction accuracy [31]. Guo et al. proposed ASTGCN based on the attention mechanism. Based on three kinds of periodic data, a spatiotemporal attention mechanism was established to effectively capture the dynamic spatiotemporal correlation in traffic data [32]. However, these methods only use the Attention mechanism for simple temporal correlation modeling, which cannot fully capture the deep information hidden in the time series. The application of the Transformer needs to be studied.

The transformer is a model based on the Seq2Seq architecture that integrates attention mechanism, location coding, residual connection, and layer standardization [33]. It can effectively extract the long-term temporal correlation hidden in the input sequence by directly calculating the attention weight of the sequence data at each position. It is suitable for sequence input of different lengths and can capture the deep temporal correlation existing in the long-term range. There is still much space for research based on Transformer. Song et al. proposed a model combining GAT and Transformer—TSTNet [34], which uses random walking to map the characteristics of GAT learning to spatial embedding and combines time embedding as a spatiotemporal sequence. Finally, it uses Transformer to extract the spatiotemporal correlation in the sequence.

## 3. Methodology

In this section, we define the research problem in the first part. Specifically, for the topological map network division of regional data, we introduce the time-space sequence and then describe the process of traffic classification and traffic prediction tasks. In the second part, we introduce the main structure of SGGformer, which is divided into three parts: time segment division based on a shifted window operation, spatial correlation modeling, and time correlation modeling.

### 3.1. Problem Definition

#### 3.1.1. Definition 1: Regionally Topological Graph Network

**Figure 1:**
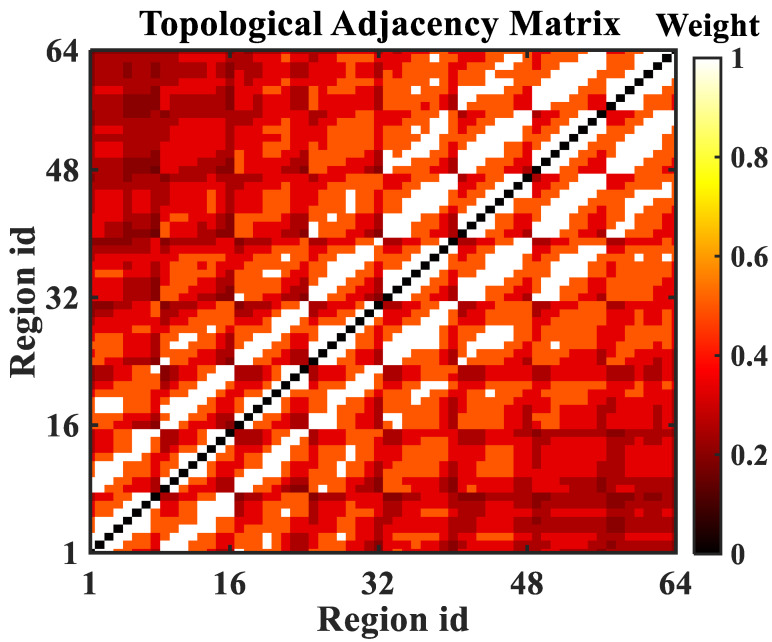
The topological adjacency matrix.

This paper describes the traffic data divided by regions in the form of a graph network. Specifically, the city’s regional data are represented as an undirected graph G=(V,E,W), where $V=\{v_{1},v_{2},\cdots ,v_{N}\}$ indicates the collection of regional nodes, *N* is the total number of regions. Moreover, an edge $e_{ij}\in E$ denotes the correlation between region $v_{i}$ and $v_{j}$. In addition, the weight $w_{ij}\in W$ of the edge $e_{ij}$ represents the degree of correlation between region $v_{i}$ and $v_{j}$, which is measured by the topological correlation between regions, and the value is equal to the reciprocal of the number of edges passed by the shortest adjacent path between regions. Specifically, there is a strong topological correlation between two regions that are separated by sell regions in the regional network, which shows a large weight in the adjacency matrix. The adjacency matrix *W* of *G* is expressed as Equation (1), and the matrix visualization is shown in Figure 1.

(1) $$W_{r}=\left[\begin{array}{cccc} 0 & \omega_{r}\left(1,2\right) & \cdots & \omega_{r}\left(1,N\right)\\ \omega_{r}\left(2,1\right) & 0 & \cdots & \omega_{r}\left(2,N\right)\\ \vdots & \vdots & \ddots & \vdots \\ \omega_{r}\left(N,1\right) & \omega_{r}\left(N,2\right) & \cdots & 0 \end{array}\right]$$

#### 3.1.2. Definition 2: Spatiotemporal Sequences

The time of a day is divided into *T* time stamps according to a certain resolution. Under each time stamp, the spatial-temporal data of the region is expressed as follows.

(2) $$S=\{X_{t}|t\in T\}$$

where *t* is time stamp, $X_{t}\in R^{N\times F}$ are the *F* traffic characteristics (average speed, average flow) in the *N* regions under the time stamp *t*. For the node $v_{i}$ in the *i*-th region in the data $X_{t}$, its traffic characteristics are expressed as $x_{n}\in R^{F}$.

#### 3.1.3. Definition 3: Traffic Grades

In order to objectively and comprehensively evaluate the traffic status, this paper takes the form of a traffic grade as the coupling representation of different traffic statuses. Specifically, different traffic statuses are coupled based on a self-organizing mapping neural network (SOM) [38,39]. The traffic grade is obtained through the training and testing process of SOM.

In the training process, firstly, the node weight vector of SOM’s output layer $W=\{\omega_{i},i=1:Cls\}$ is initialized, where Cls represents the node number which is equal to the number of traffic grades. Then, the initial learning rate l(0), initial neighborhood radius r(0), and iterations Iter are initialized. After that, the traffic sample *X* is input into the SOM, and for the sample $x_{t,n}\in R^{F},t\in T,n\in N$, the distance from all output layer nodes to them is calculated as follows:

(3) $$d_{t,n:i}\left(x\right)=\sqrt{{\left(x_{t,n}-\omega_{i}\right)}^{2}}$$

After the calculation, the nearest node is found as the winning node. The weights of the winning node and all nodes in the neighborhood are updated, as shown below.

(4) $$\omega_{j}\left(t+1\right)=\omega_{j}\left(t\right)+l\left(t\right)\times r\left(t\right)\times \left(x_{t,n}-\omega_{j}\right)$$

where j=1:Cls, and the learning rate and neighborhood radius are updated as well, which is shown as follows:

(5) $$r\left(t\right)=r\left(0\right)\times \exp\left(-d_{t,n:i}/t_{1}\right),\;t_{1}=Iter/\log\left(r\left(0\right)\right)$$

(6) $$l\left(t\right)=l\left(0\right)\times \exp\left(-t/t_{2}\right),t_{2}=Iter$$

Through continuous circulation, all traffic samples are traversed. Finally, the training will exit when the weight of the output layer does not change significantly or the maximum number of training times is reached. This paper sets the maximum number of iterations as 200 generations.

**Figure 2:**
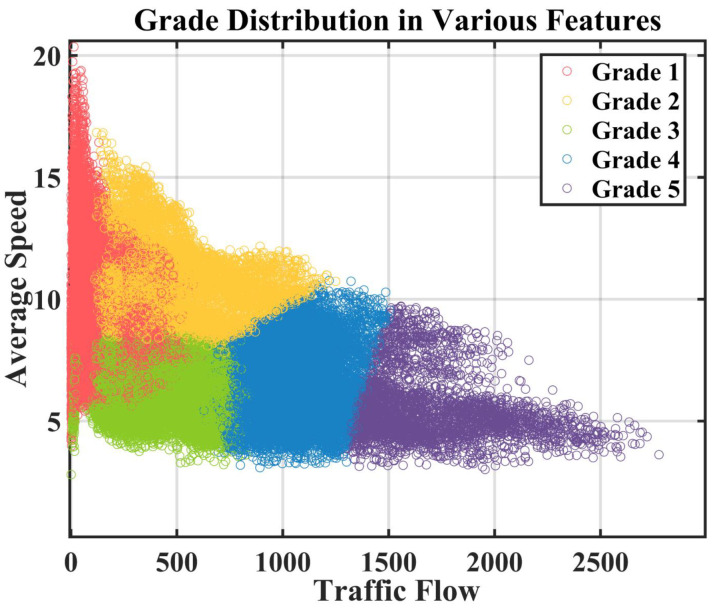
Distribution of sample points under different features after SOM clustering.

**Figure 3:**
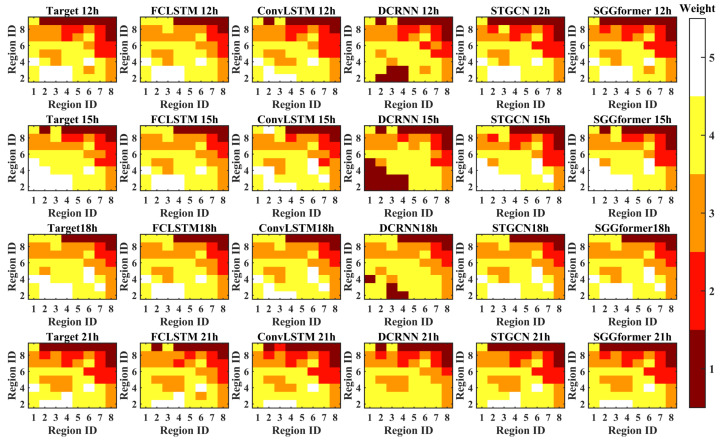
Distribution of traffic grades for 24 h of the day for the full graph in the test set.

In the test process, based on the trained SOM network parameters, the corresponding traffic grade of each regional node is obtained. Specifically, the traffic samples *X* are input into the SOM network. For each traffic sample $x_{t,n}\in R^{F},t\in T,n\in N$, we calculate the Euclidean distance between all SOM output layer nodes and the sample. The formula is as follows:

(7) $$d_{t,n:i}\left(x\right)=\sqrt{{\left(x_{t,n}-\omega_{i}\right)}^{2}}$$

where i=1…Cls. The nearest node $\omega_{i^{\prime }}$ is found to determine the traffic grade of the sample $x_{t,n}$ as $i^{\prime }$. By completing the traversal of all nodes, traffic grade samples $Y_{t}\in R^{N}$ are obtained, which represent the traffic state in *N* regions under the timestamp *t*. The distribution of regional feature points after classification is shown in Figure 2. Besides, Figure 3 shows the grade change of all regions in a certain day.

#### 3.1.4. Definition 4: Traffic Prediction Task

Based on the defined regional graph network and the traffic samples $X\in R^{W\times N\times F}$ (regional average speed, average flow) with a history of length *W* under a given times tamp *t*, the goal of the traffic prediction task is to learn a mapping function that can predict the traffic state grade under the next time stamp *h*, specifically as follows:

(8) $$Y_{t+h}=f_{\theta }\left(X_{t-W+1}\cdots X_{t-1},X_{t},G\right)$$

where θ represents the parameters of the mapping function.

### 3.2. SGGformer

**Figure 4:**
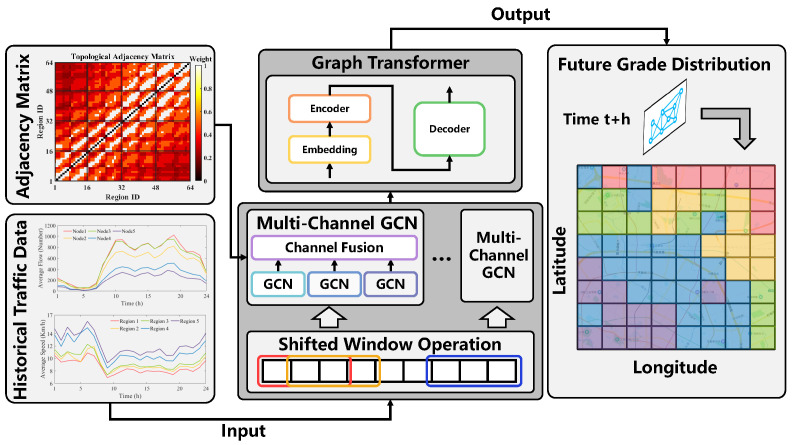
Overall architecture diagram of SSGformer. Historical traffic data is firstly divided into different segments by shifted window operation, and then multi-channel GCN operation is performed on different historical segments in parallel based on the topological adjacency matrix to obtain spatial correlation characteristics. Then, the Graph Transformer module is used to further complete the extraction of time-related features. Finally, the traffic grade of all regions is output in the whole graph corresponding to the time of practice t+h. Specifically, the Chinese characters in the regional grade map represent the names of roads and landmark sites.

The overall architecture of the SGGformer model proposed in this paper is shown in Figure 4, including three main components: shifted window operation, multi-channel GCN, and Graph Transformer. The overall model input includes the urban area’s historical 24 h characteristics (average flow and average speed) and the topological adjacency matrix. The final output is the entire graph traffic grade at the future time stamp through the characteristic extraction of the model. For the three parts of the model, first of all, the following introduces the shifted window operation of SGGformer, which divides the traffic data into historical stages according to the time dimension. Then, the process of establishing spatial dependency of traffic data by multi-channel GCN is described. Finally, the establishment of time dependency by the Graph Transformer is explained, which can directly process GCN to generate high-dimensional graph data sequences. Furthermore, the Seq2Seq architecture of the Transformer type network is utilized to complete the traffic state prediction at the future time.

#### 3.2.1. Shifted Window Operation

**Figure 5:**
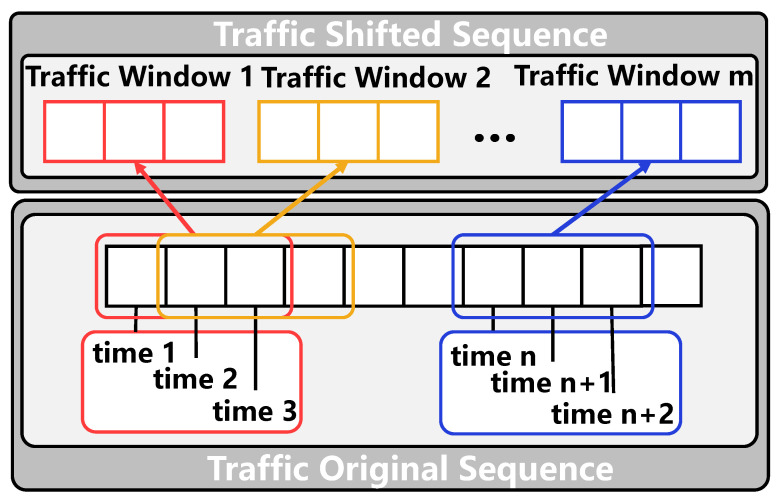
The process of the shifted window operation.

The historical traffic data often show a certain stage. That is, the traffic flow remains relatively stable for a certain period, with the arrival of the next stage, the traffic conditions show a distinct difference. It can be understood that during the morning rush hour, the roads in the urban area become crowded. As time goes by, the congestion of the roads will improve, and the traffic will deteriorate again when it reaches noon. At the same time, the time resolution is coarsened by dividing the shifted window operation, and feature extraction is performed based on the coarsened time interval in the time-dependent modeling process, which reduces the computational complexity to a certain extent. Therefore, in this paper, the historical traffic data are divided into sliding segments according to a particular time window, and the segmentation of different periods is realized according to the size and step size of the sliding window, as shown in Figure 5.

Based on the traffic data with a history length of *W* time stamps, this paper selects the sliding window size of (W/3) and the step size of (W/6), so the historical data will be divided into five time windows, which are represented as $P_{i}\in R^{N\times (W/3)\times F}$. The equation for the dividing window is as follows:

(9) $$P_{i}=\left[X_{(i-1)*(W/6)+1},...,X_{\left(i+1)*(W/6\right)}\right]$$

#### 3.2.2. Spatial Correlation Modeling

**Figure 6:**
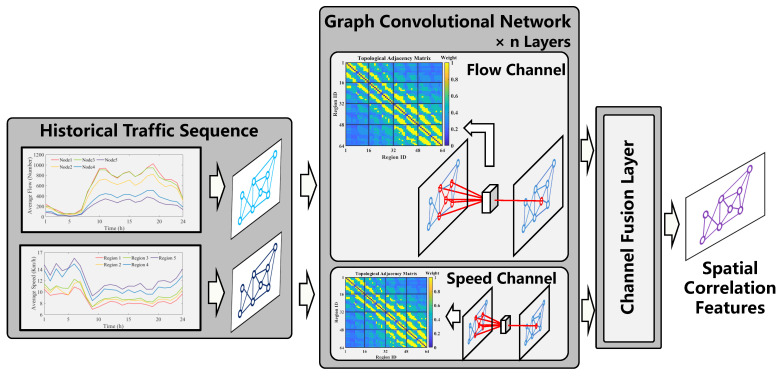
The process of spatial correlation modeling.

SGGformer establishes spatial dependencies by building a multi-channel GCN model, as shown in Figure 6. First, the segmented data of different windows are input into the stacked multi-channel GCN in parallel. Under each segment input, the parallel mechanism is also used between different channels. After extracting the local spatial features of GCN, the channel fusion layer is used to fuse the different types of spatial features captured. Specifically, this paper mainly includes two features, namely, regional flow and average speed. The overall spatial modeling process is shown in Equation (10).

(10) $$\begin{array}{c} H_{MG}=Fusion\left(GCN_{flow}^{}\left(P_{i}^{flow}\right),GCN_{speed}^{}\left(P_{i}^{speed}\right)\right)\\ =GCN_{flow}\left(P_{i}^{flow}\right)+GCN_{speed}\left(P_{i}^{speed}\right) \end{array}$$

where $P_{i}^{flow}\in R^{N\times (W/3)}$ and $P_{i}^{speed}\in R^{N\times (W/3)}$ represent the traffic data corresponding to the two characteristic dimensions of traffic flow and average speed under window *i*. GCN is proposed by Kipf et al. which is widely used in the field of traffic prediction because of its simple calculation process and effective spatial feature extraction. Specifically, based on the defined topological adjacency matrix and the combination of linear transformation and activation function, it completes the feature capture of regional nodes. The GCN is the first-order approximation of the spectral graph convolution, particularly, the aggregation and mapping of the node’s first-order neighbor features are completed by a first-order approximation of the Chebyshev polynomial, and the information transmission of the multi-order neighborhood can be realized by stacking several GCN layers. For a layer of GCN, the formula is as follows:

(11) H(k)=GCN(W(k),H(k−1);θ(k))=ReLUD˜(k)−12W˜D˜(k)−12H(k−1)θ(k)

where $\tilde{W}=W+I$ is the adjacency matrix considering the self-loop, ${\tilde{D}}^{\left(k\right)}=\sum_{j}{\tilde{W}}_{ij}^{\left(k\right)}$ represents the degree matrix, $\theta^{\left(k\right)}\in R^{d\times d^{\prime }}$ denotes a trainable parameter matrix, $W^{\left(k\right)}\in R^{N\times N}$ is the adjacency matrix of the graph network, $H^{\left(k-1\right)}\in R^{N\times d^{\left(k-1\right)}}$ is the node representation of the k−1-th layer output, and $H^{\left(k\right)}$ indicates the k−1-th layer output node representation after feature extraction. $d^{\left(k-1\right)}$ and $d^{\left(k\right)}$ represent the numbers of node features corresponding to the layer (k−1) and *k*, respectively. For the node features of the first layer input, $d^{\left(1\right)}=W/3$.

#### 3.2.3. Temporal Correlation Modeling

**Figure 7:**
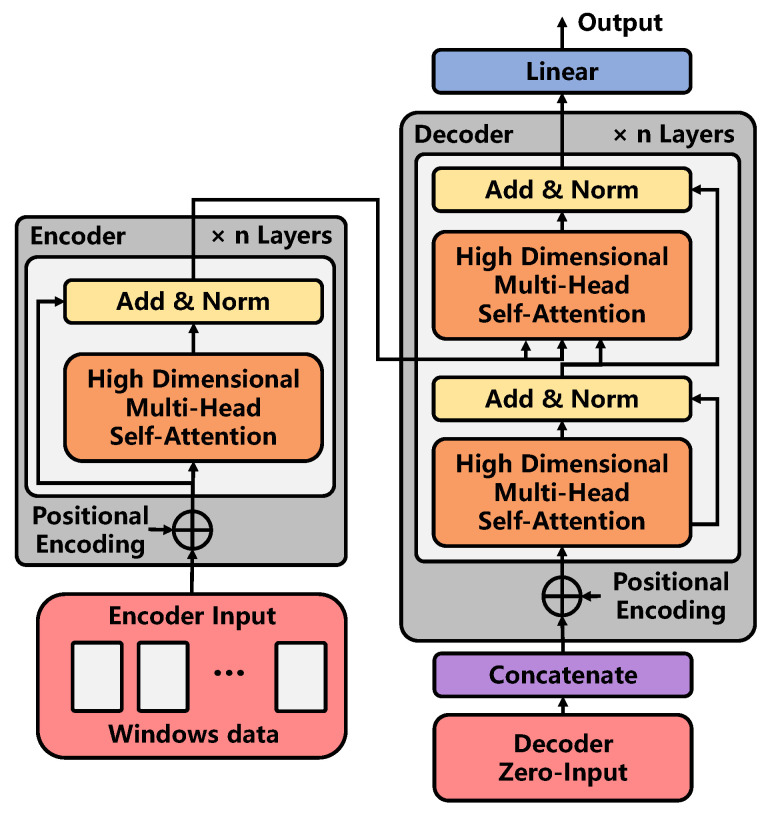
The process of temporal correlation modeling.

In this paper, the constructed Graph Transformer is used to extract the temporal-related characteristics of traffic data. Its architecture is shown in Figure 7. Graph Transformer is composed of an encoder and a decoder. It is based on the improvement of the traditional Transformer. The improvement mainly consists of the following parts:

1. The encoder and decoder are composed of high-dimensional self-attention modules with residual connections. The purpose of constructing that module is to directly extract the time characteristics of multi-dimensional graph data obtained from graph convolution;
2. In the decoder part, thanks to the inspiration of Informer [30], its generative decoder can output an extended sequence in a single step by inputting an input with zero occupation, thus avoiding cumulative error and greatly reducing the reasoning speed.

In conclusion, this paper does not make many improvements to the excellent structure of the Transformer. However, it focuses on the input form of each sub-feature extraction unit and decoder in the structure. The following will be introduced respectively from the Graph Transformer architecture: high-dimensional self-attention mechanism and zero-occupation input of the decoder.

Graph Transformer Architecture:

The architecture of the graph transformer, such as neural transformation models including the Transformer, has an encoder–decoder structure. Here, the encoder maps the high-dimensional historical features of nodes extracted by the Multi-Channel GCN layer to the continuous representation $z=\left(z_{1},\cdots z_{win}\right)\in R^{win\times n\times emb}$. Here, emb represents the embedded characteristic dimension of the node. Given *z*, the decoder is input to the matrix spliced by a partial historical feature embedding matrix and zero-occupation matrix, so as to realize the output of the spatial-temporal characteristics at the future time in one step.

Encoder: The encoder is composed of a stack of m=2 identical layers. Each layer contains a high-dimensional autonomous willpower mechanism. We use the residual connection between different layers, and then normalize the layers. The calculation formula is as follows:

(12) $$z_{enc\_out}^{\left(k\right)}=LayerNorm\left(z_{enc\_in}^{\left(k\right)}+SubLayer_{S-Attn}\left(z_{enc\_in}^{\left(k\right)}\right)\right)$$

where $z_{enc\_in}^{\left(k\right)}$ and $z_{enc\_out}^{\left(k\right)}$ represent the input and output node characteristics of the sub-layer of the layer *k*, respectively. $SubLayer_{S-Attn}\left(.\right)$ represents the high-dimensional self-attention mechanism, as shown in this section. In order to facilitate residual connection, all sub-layers and embedded layers in the model maintain dimensions emb=64 and LayerNorm(.) represents layer normalization functions.

Decoder: The decoder is also composed of n=2 stacks of the same layer. In addition to the layer in the encoder, a second layer is inserted, which performs a multi-head attention mechanism on the output of the encoder stack, which is shown in Equation (13). Similar to the encoder, residual connections are used between each layer, and then, layer normalization is performed. Similar to Transformer, we modify the self-attention layer in the decoder stack to prevent locations from focusing on subsequent locations. This mask, combined with output embedding, ensures that the prediction of *i* locations can only rely on known outputs with locations smaller than *i*.

(13) $$\left\{\begin{array}{c} z_{dec\_out}^{\left(k\right)}=LayerNorm\left(h_{dec}^{\left(k\right)}+SubLayer_{Attn}\left(z_{enc\_out}^{\left(end\right)},h_{dec}^{\left(k\right)}\right)\right)\\ h_{dec}^{\left(k\right)}=LayerNorm\left(z_{dec\_in}^{\left(k\right)}+SubLayer_{S-Attn}\left(z_{dec\_in}^{\left(k\right)}\right)\right) \end{array}\right.$$

where $z_{dec\_out}^{\left(k\right)}$ is the output of the decoder, $h_{dec}^{\left(k\right)}$ represents the intermediate variable in the decoder, $z_{enc\_out}^{\left(end\right)}$ denotes the output characteristics of the encoder at the last layer, $z_{dec\_in}^{\left(k\right)}$ indicates the zero-occupation input of the decoder, see for details in [30]. The second layer inserted is a high-dimensional attention mechanism $SubLayer_{Attn}\left(.\right)$, see below for details.

After the decoder completes the extraction of the temporal feature, the feature data are input to the linear layer to complete the mapping of the feature to all node grades. The calculation process is shown as follows:

(14) $$z_{l\_out}={\left[ReLU\left(W_{lin}^{\left(l\right)}\times z_{dec\_out}\right)+b_{lin}^{\left(l\right)}\right]}_{\times L_{linear}}$$

where ReLU(.) represents ReLU activation function, ${W_{lin}}^{\left(l\right)}\in R^{l\_in(l)\times l\_out(l)}$ and ${b_{lin}}^{\left(l\right)}\in R^{l\_out(l)}$ represents the mapping weight and offset of the layer, respectively. $L_{linear}$ is the number of stacked linear layers. Finally, the characteristics of all roads at various grades $z_{l\_out}\in R^{N\times Cls}$ are output.

High-dimensional Self-Attention Mechanism:

The conventional multi-head self-attention mechanism obtains the relative relationship between elements through the dot product so that the relative relationship between elements is completely preserved in the feature extraction process of the sequence, and all elements are processed in parallel in the calculation process to improve the calculation efficiency. Considering the high number of features of elements in the sequence, parallel computing in the form of multiple heads can extract the relationship between elements in the sequence from different angles and further increase computational efficiency. Therefore, the multi-head self-attention mechanism is widely used in the feature extraction of sequences.

**Figure 8:**
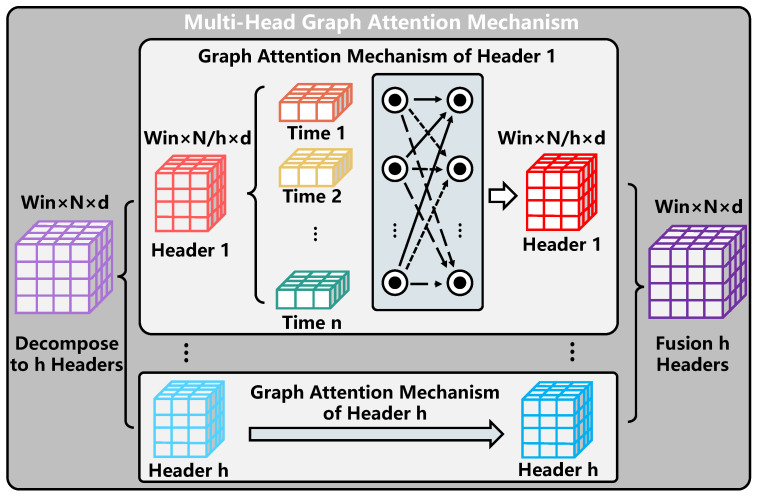
The calculation process of the high-dimensional self-attention.

In this paper, the spatial feature sequence extracted by GCN in different time windows is taken as the input, and each element in the sequence represents the spatial feature in this window. The fusion and feature extraction of spatial features in different time windows are completed using the self-attention mechanism to capture the temporal correlation among them. However, compared with the input data of the conventional self-attention mechanism, the combined sequence of different spatiotemporal features in this paper has a higher dimension. Specifically, compared with the input data of the normal multi-head voluntary mechanism, the input data of the high-dimensional multi-head self-attention mechanism are in the form of a two-dimensional matrix, which increases the dimension of the number of roads. Therefore, this paper improves the conventional attention mechanism and designs a high-dimensional multi-head self-attention mechanism, which is shown in Figure 8. The specific calculation process is as follows.

We first define the matrix of query, key, and value ($Q\in R^{win\times N\times d}$, $K\in R^{win\times N\times d}$ and $V\in R^{win\times N\times d}$), and then obtain three functional matrices by performing a linear transformation on the second dimension (i.e., node number dimension) of the embedded traffic data $X\in R^{win\times N\times d}$ and decomposing it into multiple heads, as shown in Equation (15).

(15) $$\begin{array}{c} Q_{i}={\left[reshape\left(W_{Q}X\right)\right]}_{i}\\ K_{i}={\left[reshape\left(W_{K}X\right)\right]}_{i}\\ V_{i}={\left[reshape\left(W_{V}X\right)\right]}_{i} \end{array}$$

where $W_{Q}\in R^{N\times n}$, $W_{K}\in R^{N\times N}$, and $W_{V}\in R^{N\times N}$ represent the learnable parameter matrix, *h* is the number of headers, and shape(.) is the header splitting operation of the matrix, that is, the size is converted from [win×n×d] to $\left[win\times h\times \frac{N}{h}\times d\right]$. In addition, $Q_{h}\in R^{win\times \frac{N}{h}\times d}$, $K_{h}\in R^{win\times \frac{N}{h}\times d}$, and $V_{h}\in R^{win\times \frac{N}{h}\times d}$ represent the three functional matrices corresponding to the *i*-th header obtained through linear mapping and the splitting operation, respectively.

Then, we further use the dot product attention operation to represent the high-dimensional self-attention layer, as shown in the following.

(16) $$a_{i}^{t,t^{\prime }}=soft\max\left(f\left(Q_{i}^{t},K_{i}^{t^{\prime }}\right)\right)=\frac{\exp(f\left(Q_{i}^{t\times (d_{q}\times d)},K_{i}^{t^{\prime }\times \left(d_{k}\times d\right)}\right))}{\sum_{t^{\prime }\in T_{p}}\exp(f\left(Q_{i}^{t\times (d_{q}\times d)},K_{i}^{t^{\prime }\times \left(d_{k}\times d\right)}\right))}$$

(17) $$head_{i}=Attention\left(Q_{i},K_{i},V_{i}\right)=\sum_{t\in T_{p}}a_{i}V_{i}^{t}\;i\in \left[1,\cdots ,h\right]$$

(18) $$X_{fc}=MultiHead\left(Q,K,V\right)=Concat\left(head_{1},\cdots ,head_{h}\right)W^{O}$$

where $a_{t,t^{\prime }}\in A\in R^{d}\left(A\in R^{T_{p}\times T_{p}\times d}\right)$ represents the normalized weight matrix between combination *t* and combination $t^{\prime }$, $t\in T_{p},t^{\prime }\in T_{p}$, and the second dimension is equal to $d_{q}=d_{k}=d_{v}=\frac{N}{h}$. $head_{i}$ represents the characteristic matrix under the *i*-th header. Finally, the final output of the high-dimensional multi-head self-attention mechanism is obtained by splicing and linear mapping of all Matrices corresponding to all header matrices.

The difference between the high-dimensional attention mechanism H−DAttn(.) and the high-dimensional self-attention mechanism is reflected in the input. The query, key, and value of the high-dimensional attention mechanism can be different inputs. Three functional matrices can be obtained by performing a linear transformation on the second dimension (i.e., node number dimension) of different traffic-embedded data $X_{1}\in R^{win_{1}\times n\times d},X_{2}\in R^{win_{2}\times n\times d},X_{3}\in R^{win_{3}\times n\times d}$ and decomposing them into multiple heads, as shown in the formula below.

(19) $$\begin{array}{c} Q_{i}={\left[reshape\left(W_{Q}X_{1}\right)\right]}_{i}\\ K_{i}={\left[reshape\left(W_{K}X_{2}\right)\right]}_{i}\\ V_{i}={\left[reshape\left(W_{V}X_{3}\right)\right]}_{i} \end{array}$$

After the linear mapping operation, the corresponding *Q*, *K*, *V* matrices are obtained. The following operations are the same as the above self-attention mechanism and will not be repeated.

Decoder Zero-occupation Input:

Because the traditional Seq2Seq architecture performs multi-step prediction through continuous self-regression in the decoding process, which reduces the calculation speed in the prediction process. Referring to the experience obtained by the Informer [30], similarly, we concatenate some historical features with zero vectors as the input of the decoder, as shown in the following.

(20) $$X_{de}^{t}=Concat\left(X_{tok}^{t},X_{0}^{t}\right)\in R^{(L_{tok}+L_{y})\times N\times d}$$

where $X_{tok}^{t}\in R^{L_{tok}\times N\times d}$ is the starting token and $X_{0}^{t}\in R^{L_{y}\times N\times d}$ is the placeholder of the target sequence (set the scalar to 0), which equals the number of the prediction length. The starting token is a sequence of length sampled from the encoder input sequence, which corresponds to a segment before the prediction time. This paper takes the prediction of the next 1 h as an example, the sequence input to the encoder is the node characteristic sequence of five historical time periods. We take the last two time periods of the sequence as the starting token, namely $L_{tok}=2$, and input $X_{de}=\left\{X_{2p},X_{0}\right\}$ to the decoder. Note that the decoder here predicts all outputs in one step, and does not need time-consuming “dynamic decoding” transactions in the ordinary encoder–decoder architecture, which greatly reduces the calculation time.

#### 3.2.4. Loss Function

SGGformer uses Negative Log Likelihood Loss (NLLLoss) as the target loss function. After the feature extraction of the spatiotemporal network, the matrix $X_{out}\in R^{N\times Cls}$ representing all regions at different levels is obtained. Before calculating the loss value, it is necessary to calculate the probability distribution of each road corresponding to different grades. Therefore, the above matrix is operated by Softmax operation, which is shown as follows:

(21) $${\hat{Y}}_{i,j}=Softmax\left(X_{i,:}\right)=\frac{\exp(X_{i,j})}{\sum_{k}\exp(X_{i,k})}$$

where $X_{i,j}$ refers to the value of the corresponding grade *j* of the road *i* in the matrix, and the grade probability matrix $\hat{Y}\in R^{N\times Cls}$ represents the probability of all roads corresponding to different grades, which is used as the input of the loss function. The loss value is calculated as follows:

(22) $$L\left(\hat{Y},Y\right)=-\sum_{n=1}^{N}Y*\log(\hat{Y})$$

where $L(\hat{Y},Y)$ is the loss value of the prediction grade and the real grade, and $Y\in R^{N\times Cls}$ represents the real level of all roads. For each road (each line), the index value corresponding to the real grade is 1, and the rest is 0. Finally, the total loss value is obtained by summing the loss values of all roads.

## 4. Experiment

### 4.1. Data Description and Preprocessing

**Figure 9:**
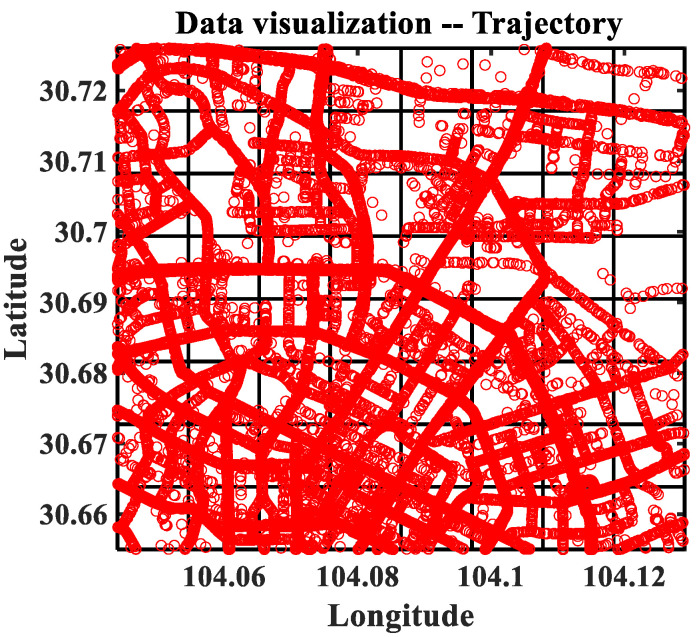
The raw trajectory data of the floating vehicles.

This experiment is based on the floating car data of a big data platform DiDi, and the vehicle track data from 1 November to 30 November 2016, are selected as the source data, which is shown as Figure 9. According to the divided sub-regions, the average speed and the average flow of the sub-regions are counted per hour, the details are described as follows.

In this paper, the track data point x is preprocessed by three main steps. First, we divide the region with the size of 8 × 8 based on the rectangular distribution range of track points. Next, the track data point is counted and calculated. The original track data point $TP_{i}=\left\{ts_{i},lon_{i},lat_{i}\right\},i\in \left\{1,\ldots ,N_{TP}\right\}$ is obtained, where $ts_{i}$ is the time stamp of track point *i*, $lon_{i}$ and $lat_{i}$ are the longitude and latitude of track point *i*, respectively. We can obtain the velocity characteristics of each track point by calculating the ratio of the distance between adjacent track points of vehicles and the time stamp. Finally, the regional characteristics are counted. For the regional flow and average speed, we can get the weighted average of the number of characteristic points and the speed of characteristic points at the corresponding time stamp.

According to the obtained regional data, we need to further develop the input data. Since this paper takes the 24 h historical regional traffic characteristics of the whole graph as the input, a total of 720 sets of datasets are obtained through the rolling selection method. Moreover, the specific parameters of the model and training process are shown in Table 1.

### 4.2. Assessment Indicators and Baseline Model

In this paper, accuracy and the quadratic-weighted Kappa coefficient (Kappa coefficient) are used as the evaluation indicators of the prediction effect. The quadratic-weighted Kappa coefficient represents the consistency between the prediction grade and the actual grade distribution, representing the prediction’s accuracy and deviation. The calculation process is based on the confusion matrix, and the value is between −1 and 1. The closer the value is to 1, the higher the consistency of the prediction grade results. Specifically, the calculation method of accuracy and weighted kappa coefficient are shown as follows.

(23) $$ACC=\frac{1}{n}\sum_{t=1}^{n}\left(1,\;ifv_{t}={\tilde{v}}_{t}\;else\;0\right)$$

(24) $$KAPPA=\frac{P_{o}-P_{e}}{1-P_{e}}$$

(25) $$\left\{\begin{array}{c} P_{o}=\sum_{i=1}^{Cls}\sum_{j=1}^{Cls}\omega_{i,j}p_{i,j}\\ P_{e}=\sum_{i=1}^{Cls}\sum_{j=1}^{Cls}\omega_{i,j}p_{i,:}p_{:,j}\\ \omega_{i,j}=1-{\left(\frac{i-j}{Cls-1}\right)}^{2} \end{array}\right.$$

where, for the accuracy rate equation, $v_{t}$ is the actual grade, ${\tilde{v}}_{t}$ is the prediction grade, *n* is the number of prediction grades. For the quadratic-weighted kappa coefficient equation, *p* is the confusion matrix, $p_{i,j}$ represents the frequency of occurrence of the data instances that the road with a real grade *i* is judged a grade *j* in all prediction results. $p_{i,:}$ represents the ratio of the number of data instances with a real grade *i* to the the total number of instances, $p_{:,j}$ represents the ratio of the number of data instances that the road is predicted as a grade *j* to the total number of instances, $\omega_{i,j}$ is a weight and Cls represents the number of grades.

To evaluate SGGformer’s competitive performance, we compare it with the following baseline. In these baselines, the FC-LSTM, ConvLSTM, DCRNN, and STGCN inputs are the average road speed or flow. All baselines are optimized for optimal performance.

FC-LSTM: A classical cyclic network used for time series data modeling. Here, the full connection layer maps the time dimension linearly. Precisely, the dimensions are mapped to 64 and 24, respectively, and then input to the double-layer LSTM layer, and finally output the final result through the two full connection layers. The hidden layer dimension of the first full connection layer is 64, the hidden layer dimension of the double layer LSTM is 64, the hidden layer dimension of the final full connection layer is 24, and the output layer dimension is 5.

ConvLSTM: A classical hybrid neural network used for traffic spatiotemporal data modeling. Here, we first model the spatial correlation of traffic data through the stacked two-layer CNN, then input it into the two-layer LSTM layer, and finally output the final result through the two-layer full connection layer. The convolution kernel size of the first layer convolution is 8, the step size is 4, the convolution kernel size of the second layer convolution is 3, the step size is 2, the hidden layer dimension of the double-layer LSTM is 64, the hidden layer dimension of the final fully connected layer is 24, and the output layer dimension is 5.

DCRNN: It refers to the method of [20], builds a directed graph based on sensors, and gives an edge weight by measuring the proximity between sensor pairs. The dynamic traffic flow is modeled as a two-way diffusion process. Diffusion convolution is proposed to capture spatial dependence, and a cyclic neural network is used to model the time dependence. The first two layers of diffusion convolution raise the dimension to 64, and the third layer of diffusion convolution reduces the dimension to 32.

STGCN: It refers to the method of [21], which combines graph and gated time convolution to learn spatiotemporal patterns from traffic sequence data based on a graph structure. The structure consists of two layers of spatiotemporal graph convolution modules. Each module consists of a layer of temporal convolution module, a layer of spatial graph convolution module, and a layer of temporal convolution module. The first layer of the time convolution module increases the number of feature channels to 64, and the space map convolution module reduces the number of feature channels to 32, the last layer of the time convolution module increases the number of feature channels to 64, and the hidden layer dimension of the last full connection layer is 80.

### 4.3. Comparison with Baseline

**Figure 10:**
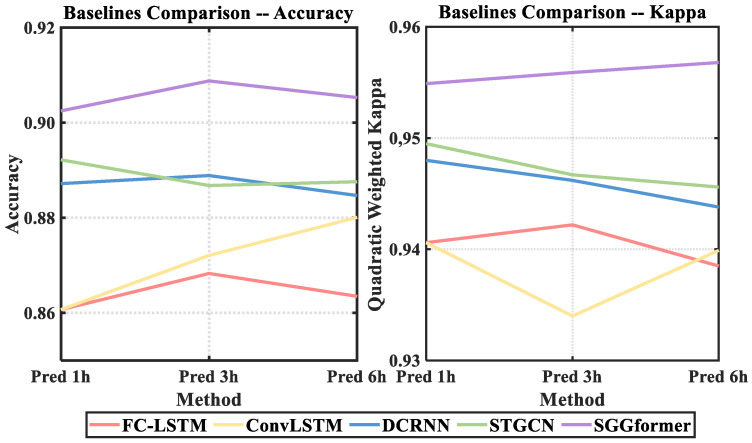
The performance comparison among SGGformer and baselines.

Figure 10 shows the traffic prediction performance between the SGGformer model and the baseline on the dataset. See Table 2 and Table 3 for specific values. Firstly, the SGGformer presented in this paper shows stable and relatively optimal tables under different prediction lengths. Based on the average performance of the three prediction lengths, the comparison accuracy and quadratic-weighted kappa coefficients are optimized by 1.7% and 0.9%, respectively, compared with the optimal baseline, and the optimization effect becomes more and more evident with the increase of prediction lengths. At the same time, the effect fluctuation of the SGGfromer under different prediction lengths is less than 1%, which proves the stability and robustness of this method under different prediction lengths.

Secondly, by comparing different deep learning baselines, it can be found that the performance of different methods on the quadratic-weighted kappa coefficient is not different, which is about 0.94. For prediction accuracy, STGCN is optimal under different lengths, and its advantages are more concentrated on short-term prediction. Specifically, with the increase in the prediction length, the prediction accuracy is reduced from 5.64% to 2.48% compared with other baselines. DCRNN is on a par with STGCN in terms of its performance different from the measured length. Specifically, its accuracy in predicting the future 1 h and 6 h groups decreases by 0.4% on average, and its accuracy in predicting the future 3 h groups exceeds 0.3%. The performance of ConvLSTM under different prediction lengths is slightly worse than that of STGCN, and the gap gradually narrows with the increase of prediction lengths, with differences of 3.15%, 1.47%, and 0.75%, respectively. Overall, FC-LSTM showed a stable prediction effect but was weaker than ConvLSTM, with an average difference of 0.68%. The gap becomes more significant with the increase of prediction length.

In order to show the prediction effects of different methods more intuitively, the figure below shows the difference between the prediction level and the actual level of different methods in the selected period when the prediction length is 1 h, 3 h, and 6 h. The periods selected in this paper are 12, 15, 19, and 22 h lower on the first day of the test set. This is because the level distribution in these periods is relatively complex.

**Figure 11:**
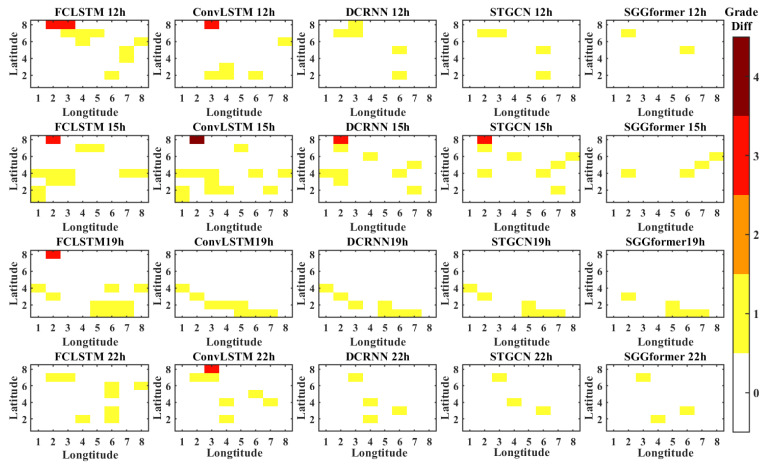
The grade difference under prediction length 1 h.

Figure 11 shows the grade difference heat map with a prediction length of 1 h. It can be found that the SGGformer shows the most accurate effect both in terms of quantity and difference. In some periods, STGCN and DCRNN had a slight difference (12, 19, and 22 h), but in some periods (15 h), the difference between the baseline method and SGGformer was noticeable. In general, the SGGformer has shown stable and excellent results in different periods. Compared with different baselines, the difference between DCRNN and STGCN is insignificant, and the effect of the ConvLSTM is worse than the two baselines. However, the distribution between the three is similar. FCLSTM has the worst effect among all comparison methods, and its distribution is not similar to other methods.

**Figure 12:**
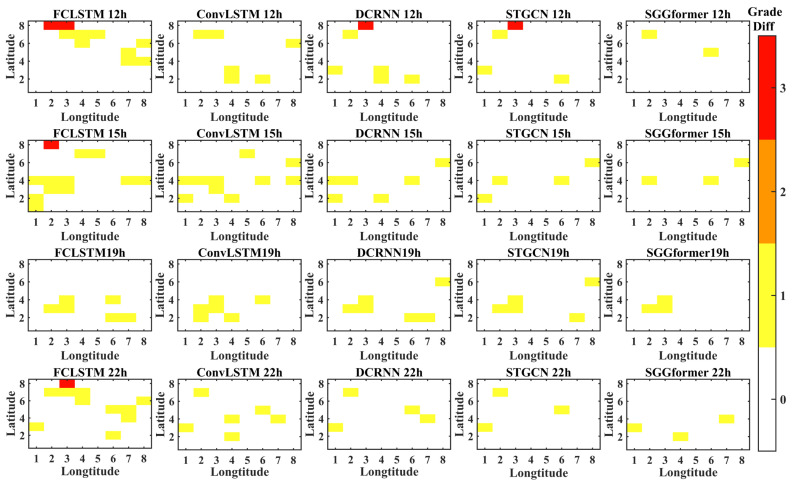
The grade difference under prediction length 3 h.

Figure 12 shows the thermal diagram of the difference between the predicted and actual grades with the predicted length of 3 h. On the whole, compared with the prediction length of 1 h, there are fewer areas of error prediction, which is consistent with the results of the prediction accuracy table above. Similarly, comparing different methods, we can find that the SGGformer still shows the most robust performance, DCRNN and STGCN show slightly worse performance, and ConvLSTM and FCLSTM show even worse performance, specifically, more error areas are predicted.

**Figure 13:**
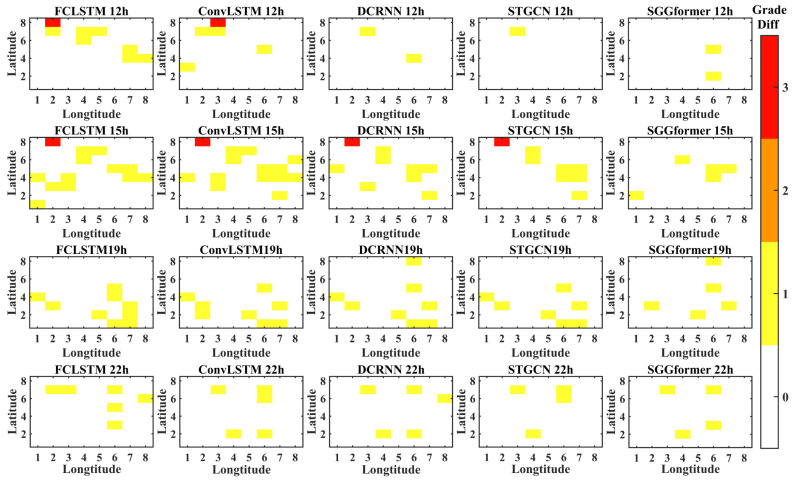
The grade difference under prediction length 6 h.

Finally, the SGGformer’s performance is optimal when the future prediction length is 6 h, which is shown in Figure 13. However, its effect is even worse than STGCN in some specific periods (such as 12 h periods). The performance of other baselines is analyzed as above.

### 4.4. Ablation Research

To study the effects of different components in the proposed SGGformer model, we repeatedly removed one of the components to conduct ablation research.

1. Based on SGGformer, the shifted window operation is removed, specifically, GCN combines Graph Transformer to build a model to verify the rationality of the shifted window;
2. The GCN based on SGGformer is removed, specifically, the GCN is directly input into the Graph Transformer network after the shifted window to verify the effectiveness of GCN for spatial feature capture;
3. The Graph Transformer based on the SGGformer is detached, specifically, SGCN is simply utilized to verify the rationality of the Graph Transformer.

**Figure 14:**
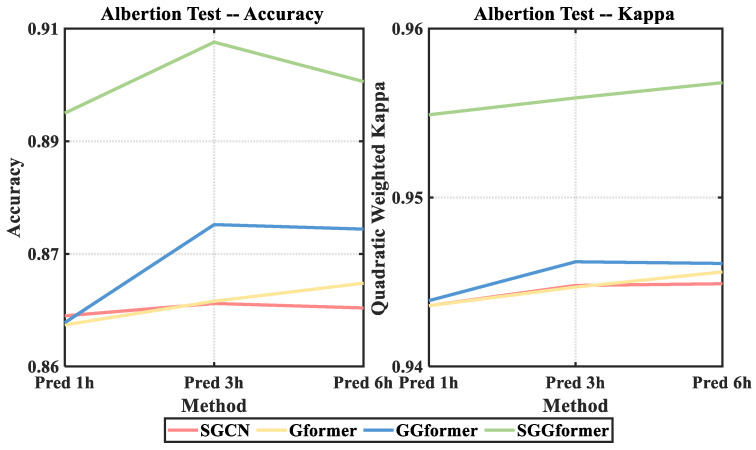
Comparison of results of ablation experiments.

Figure 14 and Table 4 and Table 5 show the accuracy and quadratic-weighted kappa coefficients of the SGGformer and its variants at different prediction lengths. For precision performance, the SGGformer has an average increase of 1.59% compared with the variant with the best performance. Among many variants, the G-Gformer has a slight advantage over the other two, showing an average increase of 0.47% in accuracy. The remaining two variants, S-Gformer and S-GCN, have similar effects when the prediction length is 1 h and 3 h, and the S-Gformer has an increase of about 0.3% compared with S-GCN when the prediction length is 6 h.

For the quadratic-weighted kappa coefficient representing consistency, the SGGformer has an average increase of 0.47% compared with the best performing variant G-Gformer. However, the difference between the quadratic-weighted kappa coefficients of other variants is slight, about 0.1%.

## 5. Discussion

The experiment part verifies the effectiveness of SGGformer in two parts: baseline comparison and ablation analysis. According to the experimental results, we can draw the following conclusions.

1. It can be seen from the baseline comparison results that the graph convolution based on the sliding window combined with Graph Transformer is effective. Precisely, graph convolution can effectively capture the non-European spatial correlation characteristics in the traffic data, and Graph Transformer has greater advantages in capturing nonlinear temporal correlation of the traffic data. STGCN, also a spatiotemporal property acquisition network based on a graph convolution network, shows a slightly weaker performance, indicating that GCN is more appropriate in spatial correlation properties. However, compared with the use of a Graph Transformer to capture temporal-related characteristics, STGCN and DCRNN use Gated convolution and GRU to extract features, respectively, which shows some shortcomings in performance. Compared with the former, ConvLSTM and FC-LSTM both use the capture time characteristics of LSTM, showing poorer prediction performance. The difference between the two is that the former uses ordinary convolution to capture European spatial characteristics. In contrast, the latter uses a simple, fully connected network to capture spatial correlation characteristics. Simply flattening regions undoubtedly ignores the spatial relationship between regions, resulting in a relatively worse prediction effect.
2. The comparison results of ablation experiments verify the effectiveness of different components of SGGformer. The G-Gformer cancels the shifted window operation, the S-Gformer deletes the GCN model and extracts the temporal-dependent characteristics through the Graph Transformer, while S-GCN uses the spatial feature extraction model for traffic prediction. The prediction effect of all variants is worse than that of all SGGformers, which proves that the three components contribute to improving the prediction effect. Among them, the prediction effect of the G-Gformer is second only to SGGformer in prediction performance, which is reasonable. Because this variant only removes the operation of the shifted window operation, it can still effectively extract spatial and temporal correlations in traffic data. However, it lacks the division of time phase by the shifted window, thus reducing specific performances. The S-Gformer and S-GCN are canceled, respectively, from the space extraction module and time extraction module, which significantly reduces the feature extraction ability for traffic data, thus showing the worst performance. By comparing the two, the S-Gformer is slightly better than S-GCN, which means that the time feature extracted by the Graph Transformer is more effective than the spatial feature extracted by GCN for traffic prediction.

Based on the above analysis, we know the advantages of the SGGformer and the necessity of different components. However, the current method of testing has some shortcomings:

1. In this paper, the spatial feature extraction network, GCN, uses a fixed adjacency matrix to extract spatial features and does not fully consider the impact of time-varying traffic flow on space.
2. This paper mainly finds that the performance of the Seq2Seq architecture is not fully utilized for traffic grade prediction at a particular time in the future. That is, the multi-step prediction task in the future is not involved.

Therefore, using a dynamic adjacency matrix and multi-step prediction is the future direction of improvement. To achieve a dynamic adjacency matrix, we consider adding an attention mechanism to capture the relative relationship of dynamic space.

## 6. Conclusions

In this paper, the sliding window operation, multi-channel graph convolution, and improved Graph Transformer are combined to extract the spatiotemporal correlation characteristics of traffic and complete the task of traffic grade prediction. The multi-channel graph convolution is used to complete the modeling of spatial dependency, and the graph transformer is used to capture the temporal dependency. Then, in terms of accuracy and prediction consistency, this paper compares the proposed SGGformer with mainstream baseline methods. The SGGformer shows better results in both aspects, verifying the effectiveness of this method. At the same time, according to the comparison results with different variants, the positive contributions of different components to the spatiotemporal feature extraction are verified.

Although the current method has achieved good results, there is still room for improvement in capturing spatiotemporal correlation. Specifically, using an attention mechanism to construct a dynamic adjacency matrix is an effective improvement method to improve the temporal extraction ability. At the same time, using Graph Transformer based on the Seq2Seq architecture to conduct multi-step prediction is also a hot topic in traffic prediction. In future work, we will integrate these improvements to improve the model’s comprehensiveness and robustness and achieve a higher prediction performance.

## Figures and Tables

**Table 1:** Hyperparameter setting for Environment Perceiver.

Detailed Parameter Setting			
Number of roads	64	Optimizer	ADAM
features of roads	3	Learning rate	5 × 10${}^{-4}$
Historical Length	24	Weight decay	1 × 10${}^{-3}$
GCN layer $L_{GCN}$	2	Batch size	24
GCN hidden Dimension	32	Training Epoch	500
Linear hidden Dimension $l_{o}ut$	64	Training set size	576
Linear layer $L_{linear}$	2	Validation set size	72
Number of traffic condition grades	5	Testing set size	72

**Table 2:** Results of comparison with the baselines on accuracy performance.

Method/ Prediction Length	1 h	3 h	6 h
FC-LSTM	0.8602	0.8683	0.8635
ConvLSTM	0.8607	0.8721	0.8801
DCRNN	0.8872	0.8889	0.8847
STGCN	0.8922	0.8868	0.8876
SGGformer	0.9025	0.9088	0.9053

**Table 3:** Results of comparison with the baselines on quadratic-weighted kappa coefficient.

Method/ Prediction Length	1 h	3 h	6 h
FC-LSTM	0.9399	0.9422	0.9385
ConvLSTM	0.9406	0.934	0.9399
DCRNN	0.9480	0.9462	0.9438
STGCN	0.9495	0.9467	0.9456
SGGformer	0.9549	0.9559	0.9568

**Table 4:** The ablation analysis on accuracy performance.

Method/ Prediction Length	1 h	3 h	6 h
S-GCN	0.8845	0.8856	0.8852
S-Gformer	0.8837	0.8858	0.8874
G-Gformer	0.8839	0.8926	0.8922
SGGformer	0.9025	0.9088	0.9053

**Table 5:** The ablation analysis on quadratic-weighted kappa coefficient.

Method/ Prediction Length	1 h	3 h	6 h
S-GCN	0.9436	0.9448	0.9449
S-Gformer	0.9436	0.9447	0.9456
G-Gformer	0.9439	0.9462	0.9461
SGGformer	0.9549	0.9559	0.9568

## Data Availability

Not applicable.

## Abbreviations

The following abbreviations are used in this manuscript:

ITS	Intelligent Transportation System
CNN(s)	Convolutional Neural Network(s)
GCN(s)	Graph Convolutional Network(s)
SVM	Support Vector Machine
ANN	Artificial Neural Network
RNN	Recurrent Neural Network
LSTM	Long Short Term Memory Neural Network
GRU	Gated Recurrent Unit
NLP	Natural Language Processing
Seq2Seq	Sequence-to-Sequence
GNN	Graph Neural Network
SOM	Self-Organizing Mapping Neural Network
STGCN	Spatial Temporal Graph Convolutional Networks
DCRNN	Diffusion Convolutional Recurrent Neural Network
STSGCN	Spatial-Temporal Synchronous Graph Convolutional Network
ASTGCN	Attention Based Spatial-Temporal
GAT	Graph Attention Network
TSTNet	Sequence to Sequence Spatial-Temporal Traffic Prediction model
T-MGCN	Temporal Multi-Graph Convolutional Network

## References

[1] Zhang Y., Chu L., Ou Y., Guo C., Liu Y., Tang X. (2021). A Cyber-Physical System-Based Velocity-Profile Prediction Method and Case Study of Application in Plug-In Hybrid Electric Vehicle. IEEE Trans. Cybern..

[2] Piro G., Cianci I., Grieco L., Boggia G., Camarda P. (2014). Information centric services in Smart Cities. J. Syst. Softw..

[3] Zhang Y., Chen Z., Li G., Liu Y., Huang Y., Cunningham G., Early J. (2022). Integrated Velocity Prediction Method and Application in Vehicle-Environment Cooperative Control Based on Internet of Vehicles. IEEE Trans. Veh. Technol..

[4] Niu K., Cheng C., Chang J., Zhang H., Zhou T. (2019). Real-Time Taxi-Passenger Prediction with L-CNN. IEEE Trans. Veh. Technol..

[5] Qiu J., Du L., Zhang D., Su S., Tian Z. (2020). Nei-TTE: Intelligent Traffic Time Estimation Based on Fine-Grained Time Derivation of Road Segments for Smart City. IEEE Trans. Ind. Informatics.

[6] Lv Z., Lou R., Singh A.K. (2021). AI Empowered Communication Systems for Intelligent Transportation Systems. IEEE Trans. Intell. Transp. Syst..

[7] Xu F., Lin Y., Huang J., Wu D., Shi H., Song J., Li Y. (2016). Big Data Driven Mobile Traffic Understanding and Forecasting: A Time Series Approach. IEEE Trans. Serv. Comput..

[8] Zhao J., Sun S. (2016). High-Order Gaussian Process Dynamical Models for Traffic Flow Prediction. IEEE Trans. Intell. Transp. Syst..

[9] Xu Y., Kong Q.J., Klette R., Liu Y. (2014). Accurate and Interpretable Bayesian MARS for Traffic Flow Prediction. IEEE Trans. Intell. Transp. Syst..

[10] Liu F., Wei Z.C., Huang Z.S., Lu Y., Hu X.G., Shi L. A Multi-Grouped LS-SVM Method for Short-term Urban Traffic Flow Prediction. Proceedings of the IEEE Conference and Exhibition on Global Telecommunications (GLOBECOM).

[11] Oh S.d., Kim Y.j., Hong J.s. (2015). Urban Traffic Flow Prediction System Using a Multifactor Pattern Recognition Model. IEEE Trans. Intell. Transp. Syst..

[12] Toncharoen R., Piantanakulchai M. Traffic State Prediction Using Convolutional Neural Network. Proceedings of the International Joint Conference on Computer Science and Software Engineering.

[13] Yao H., Tang X., Wei H., Zheng G., Li Z. (2019). Revisiting Spatial-Temporal Similarity: A Deep Learning Framework for Traffic Prediction. arXiv.

[14] Wang B., Mohajerpoor R., Cai C., Kim I., Vu H.L. (2021). Traffic4cast—Large-scale Traffic Prediction using 3DResNet and Sparse-UNet. arXiv.

[15] Jiang W., Luo J. (2022). Graph neural network for traffic forecasting: A survey. Expert Syst. Appl..

[16] Ye J., Zhao J., Ye K., Xu C. (2022). How to Build a Graph-Based Deep Learning Architecture in Traffic Domain: A Survey. IEEE Trans. Intell. Transp. Syst..

[17] Defferrard M., Bresson X., Vandergheynst P. (2016). Convolutional Neural Networks on Graphs with Fast Localized Spectral Filtering. arXiv.

[18] Atwood J., Towsley D. Diffusion-Convolutional Neural Networks. Proceedings of the 30th Conference on Neural Information Processing Systems (NIPS 2016).

[19] Kipf T.N., Welling M. Semi-Supervised Classification with Graph Convolutional Networks. Proceedings of the ICLR 2016.

[20] Yu B., Yin H., Zhu Z. (2017). Spatio-Temporal Graph Convolutional Networks: A Deep Learning Framework for Traffic Forecasting. arXiv.

[21] Li Y., Yu R., Shahabi C., Liu Y. (2017). Diffusion Convolutional Recurrent Neural Network: Data-Driven Traffic Forecasting. arXiv.

[22] Song C., Lin Y.F., Guo S.N., Wan H.Y., Intelligence A.A.A. (2020). Spatial-Temporal Synchronous Graph Convolutional Networks: A New Framework for Spatial-Temporal Network Data Forecasting. AAAI.

[23] Wu Z.H., Pan S.R., Long G.D., Jiang J., Zhang C.Q. (2019). Graph WaveNet for Deep Spatial-Temporal Graph Modeling. arXiv.

[24] Guo K., Hu Y., Qian Z., Liu H., Zhang K., Sun Y., Gao J., Yin B. (2021). Optimized Graph Convolution Recurrent Neural Network for Traffic Prediction. IEEE Trans. Intell. Transp. Syst..

[25] Xiao Y., Yin Y. (2019). Hybrid LSTM Neural Network for Short-Term Traffic Flow Prediction. Information.

[26] Tian Y., Zhang K., Li J., Lin X., Yang B. (2018). LSTM-based traffic flow prediction with missing data. Neurocomputing.

[27] Zhao Z., Chen W., Wu X., Chen P.C.Y., Liu J. (2017). LSTM network: A deep learning approach for short-term traffic forecast. IET Intell. Transp. Syst..

[28] Fu R., Zhang Z., Li L. Using LSTM and GRU neural network methods for traffic flow prediction. Proceedings of the 2016 31st Youth Academic Annual Conference of Chinese Association of Automation (YAC).

[29] Xu M., Dai W., Liu C., Gao X., Lin W., Qi G.J., Xiong H. (2020). Spatial-Temporal Transformer Networks for Traffic Flow Forecasting. arXiv.

[30] Zhou H.Y., Zhang S.H., Peng J.Q., Zhang S., Li J.X., Xiong H., Zhang W.C., Intelligence A.A.A. (2021). Informer: Beyond Efficient Transformer for Long Sequence Time-Series Forecasting. arXiv.

[31] Bai J., Zhu J., Song Y., Zhao L., Hou Z., Du R., Li H. (2021). A3T-GCN: Attention Temporal Graph Convolutional Network for Traffic Forecasting. ISPRS Int. J. Geo-Inf..

[32] Guo S., Lin Y., Feng N., Song C., Wan H. Attention Based Spatial-Temporal Graph Convolutional Networks for Traffic Flow Forecasting. Proceedings of the National Conference on Artificial Intelligence.

[33] Vaswani A., Shazeer N., Parmar N., Uszkoreit J., Jones L., Gomez A.N., Kaiser L., Polosukhin I. (2017). Attention Is All You Need. arXiv.

[34] Song X.Z., Wu Y., Zhang C.H. TSTNet: A Sequence to Sequence Transformer Network for Spatial-Temporal Traffic Prediction. Proceedings of the International Conference on Artificial Neural Networks.

[35] Lv M., Hong Z., Chen L., Chen T., Zhu T., Ji S. (2021). Temporal Multi-Graph Convolutional Network for Traffic Flow Prediction. IEEE Trans. Intell. Transp. Syst..

[36] Cao J., Guan X., Zhang N., Wang X., Wu H. (2020). A Hybrid Deep Learning-Based Traffic Forecasting Approach Integrating Adjacency Filtering and Frequency Decomposition. IEEE Access.

[37] Zhou X., Zhang Y., Li Z., Wang X., Zhao J., Zhang Z. (2022). Large-scale cellular traffic prediction based on graph convolutional networks with transfer learning. Neural Comput. Appl..

[38] Gu Y., Wang Y., Dong S. (2020). Public Traffic Congestion Estimation Using an Artificial Neural Network. ISPRS Int. J. Geo-Inf..

[39] Zhao S., Xiao Y., Ning Y., Zhou Y., Zhang D. (2021). An Optimized K-means Clustering for Improving Accuracy in Traffic Classification. Wirel. Pers. Commun..

